# Supplementary material for: HIVprotI: an integrated web based platform for prediction and design of HIV proteins inhibitors
Source: J Cheminform. 2018 Mar 9;10:12. doi: 10.1186/s13321-018-0266-y (PMC5845081; doi:10.1186/s13321-018-0266-y)
Supplement: Supplementary file 2 — Additional file 2. Source code of HIVProtI web server. [file 13321_2018_266_MOESM2_ESM.zip › HIVprotI_Source-code/hivprotI/weka-3-6-6/doc/weka/classifiers/evaluation/package-summary.html]

weka.classifiers.evaluation


---


|  |  |  |
| --- | --- | --- |
| **Overview** \| **Package** \| Class \| **Tree** \| **Deprecated** \| **Index** \| **Help** \| **Weka's home** | |  |
| **PREV PACKAGE**   **NEXT PACKAGE** | **FRAMES**    **NO FRAMES**     **All Classes** |  |


---

## Package weka.classifiers.evaluation

| **Interface Summary** | |
| --- | --- |
| **Prediction** | Encapsulates a single evaluatable prediction: the predicted value plus the actual class value. |

| **Class Summary** | |
| --- | --- |
| **ConfusionMatrix** | Cells of this matrix correspond to counts of the number (or weight) of predictions for each actual value / predicted value combination. |
| **CostCurve** | Generates points illustrating probablity cost tradeoffs that can be obtained by varying the threshold value between classes. |
| **EvaluationUtils** | Contains utility functions for generating lists of predictions in various manners. |
| **MarginCurve** | Generates points illustrating the prediction margin. |
| **NominalPrediction** | Encapsulates an evaluatable nominal prediction: the predicted probability distribution plus the actual class value. |
| **NumericPrediction** | Encapsulates an evaluatable numeric prediction: the predicted class value plus the actual class value. |
| **ThresholdCurve** | Generates points illustrating prediction tradeoffs that can be obtained by varying the threshold value between classes. |
| **TwoClassStats** | Encapsulates performance functions for two-class problems. |

---


|  |  |  |
| --- | --- | --- |
| **Overview** \| **Package** \| Class \| **Tree** \| **Deprecated** \| **Index** \| **Help** \| **Weka's home** | |  |
| **PREV PACKAGE**   **NEXT PACKAGE** | **FRAMES**    **NO FRAMES**     **All Classes** |  |


---
